# Endocervical Curettage and Extended HPV Genotyping as Predictors of Residual Disease After Hysterectomy in Postmenopausal Women Previously Treated with LEEP for CIN3: A Multivariate Analysis

**DOI:** 10.3390/cancers17132264

**Published:** 2025-07-07

**Authors:** Maria Teresa Bruno, Antonino Giovanni Cavallaro, Maria Fiore, Zaira Ruggeri, Martina Somma, Alessia Pagana, Giuseppe Mascellino, Antonio Simone Laganà

**Affiliations:** 1 Department of General Surgery and Medical Surgery Specialties, Gynecological Clinic, University of Catania, 95123 Catania, Italy; ninocavallaro@tin.it (A.G.C.);; 2 Department of Medical, Surgical Sciences and Advanced Technologies “G.F. Ingrassia”, University of Catania, 95123 Catania, Italy; mfiore@unict.it; 3 Cervical Cancer Screening Unit, Level II, Azienda Sanitaria Provinciale di Messina, 98123 Messina, Italy; 4 Department of Experimental and Clinical Medicine, University of Catanzaro, 88100 Catanzaro, Italy; 5 Unit of Obstetrics and Gynecology, “Paolo Giaccone” Hospital, Department of Health Promotion, Mother and Child Care, Internal Medicine and Medical Specialties (PROMISE), University of Palermo, 90127 Palermo, Italy; giuseppe.mascellino@unipa.it (G.M.); antoniosimone.lagana@unipa.it (A.S.L.)

**Keywords:** CIN3, LEEP, hysterectomy, HPV genotyping, endocervical curettage, residual disease, postmenopausal, occult carcinoma

## Abstract

In postmenopausal women with high-grade cervical intraepithelial neoplasia (CIN3), hysterectomy is frequently performed after loop electrosurgical excision procedure (LEEP) due to the concern for residual disease or occult carcinoma. However, the decision to proceed with hysterectomy is often made without validated predictive criteria, increasing the risk of overtreatment or underdiagnosis. The aim of this study is to identify independent predictors of residual CIN2+ (CIN2, CIN3, adenocarcinoma in situ, invasive carcinoma) or invasive disease in hysterectomy specimens following LEEP in this high-risk population. Methods: We conducted a multicenter retrospective study including 154 postmenopausal women (aged 50–75) who underwent total hysterectomy within 12 months after LEEP for histologically confirmed CIN3. Data collected included human papillomavirus (HPV) genotyping (pre- and post-LEEP), endocervical curettage (ECC), cone margin status, transformation zone type, and histopathological outcomes of the hysterectomy specimen. Logistic regression and ROC curve analysis were used to assess predictive factors. Results: Residual disease (CIN2+, AIS, or carcinoma) was found in 38 patients (24.7%), including 7 cases (4.5%) of occult carcinoma. Persistent high-risk HPV post-LEEP was the strongest independent predictor (adjusted OR for HPV 16/18: 74.0; *p* < 0.001), followed by positive ECC (OR: 3.64; *p* = 0.028). Cone margin status was not independently associated. The multivariate model showed good discriminative performance (AUC = 0.860; sensitivity 67.2%, specificity 72.8%). Conclusions: Our findings suggest that persistent high-risk HPV infection and positive ECC are reliable predictors of residual or occult disease. These markers should be integrated into post-LEEP follow-up protocols to better identify candidates for hysterectomy and minimize unnecessary surgeries.

## Simple Summary

This retrospective study analyzed predictors of residual disease in HPV-positive postmenopausal women undergoing hysterectomy after LEEP. Statistical analysis included Cramér’s V index for strength of association, logistic regression, and ROC curve to assess predictive performance. Residual disease was found in 24.7% of cases. Although positive margins were associated with residual disease in univariate analysis, they were not confirmed in multivariate analysis, where HPV persistence and positive ECC were shown to be independent predictors. These results are clinically relevant, since they allow, on the one hand, to avoid overtreatment in low-risk patients, and on the other, to intercept occult carcinomas early on in high-risk women. It also underlines how in postmenopausal women, extended HPV genotyping is particularly useful, carefully evaluating the persistence of both 16/18 and non-16/18 genotypes, in order to personalize follow-up and therapeutic decisions.

## 1. Introduction

Human papillomavirus (HPV) infection is the most common sexually transmitted infection in the world, and high-risk oncogenic genotypes with persistence capacity are responsible for the onset of cervical carcinoma [1]. The virus also exerts its oncogenic capacity against other organs: oropharynx, anus, vulva, penis, and vagina [2,3]. According to data from the World Health Organization, cervical cancer is the fourth most common cancer among women globally, with about 600,000 new cases and more than 340,000 deaths per year [4,5].

Most cases of cervical cancer develop in women who have not had access to effective screening programs or who have not been vaccinated against HPV [6]. Previous studies have shown that postmenopausal women have a higher risk of developing occult carcinoma (10.38–17%) compared to women of reproductive age, due to several factors: difficulty visualizing the squamocolumnar junction (SCJ), squamous epithelial atrophy, and reduced accuracy of colposcopy and cytology. In contrast, in women of childbearing age, occult invasive lesions are generally less likely to be present, and conservative management may be appropriate in selected cases [7,8,9,10,11]. In particular, the transformation zone (TZ) in postmenopausal women tends to be type 3, meaning it is not visible on colposcopy, which often makes a loop electrosurgical excision procedure (LEEP) necessary for diagnostic and therapeutic purposes [12].

LEEP is considered the first-line treatment for managing high-grade Cervical Intraepithelial Neoplasia (CIN3) [13]. Although generally effective, LEEP has a risk of positive margins, viral persistence, and residual disease, which in postmenopausal women can reach higher rates than in younger patients [13,14,15,16,17,18]. Several studies have shown that in this population, colposcopic evaluation is often inconclusive and follow-up is more complex, justifying the use of hysterectomy in some circumstances [19].

However, hysterectomy is generally not recommended as a primary treatment for CIN3 according to the guidelines of the American Society for Colposcopy and Cervical Pathology (ASCCP), as it is a radical procedure with potential complications [13]. Moreover, hysterectomy as a treatment option for CIN3 should only be performed after invasive cancer has been definitively ruled out.

In this context, the identification of reliable predictive factors of residual or invasive disease is critical for proper patient selection. Several markers have been proposed in the literature: surgical margin status, HPV genotyping, endocervical curettage (ECC) outcome, and the type of transformation zone. However, there is still heterogeneity in the reported results.

The aim of the present study is to retrospectively analyze a cohort of postmenopausal women who underwent LEEP for CIN3 and later underwent hysterectomy, in order to identify the main predictors of residual disease in surgical specimens and support more appropriate treatment decisions.

## 2. Materials and Methods

We conducted a retrospective, multicenter study. We included 189 postmenopausal women, aged 50 to 75 years, who underwent hysterectomy within 12 months of LEEP for a histological diagnosis of CIN3 between 2013 and 2022.

We included patients whose medical records contained data on colposcopy, HPV testing and genotyping, endocervical curettage (ECC), LEEP, hysterectomy, and related histological examinations.

Inclusion criteria were: postmenopausal status; positive HPV test for high-risk oncogenic genotypes; colposcopy with identification of a type 3 transformation zone; and history of LEEP with ECC for HSIL, with hysterectomy performed within 12 months.

Exclusion criteria were: history of previous LEEP and incomplete clinical or histological data. Clinical characteristics of the patients were collected from the dedicated and anonymized database and included: age, type of transformation zone (TZ), status of LEEP margins, findings of ECC, pre- and post-LEEP HPV testing, including genotyping, and histopathological diagnosis from the hysterectomy specimen.

The Ethics Committee of the University Hospital waived the requirement for ethical approval and informed consent, as the archived data were used in accordance with the current legislation (20 March 2008), AIFA.

### 2.1. HPV Test and Genotyping

After cytological sampling for HPV DNA, the samples were sent to the laboratory for DNA extraction and genotyping of viral DNA by genetic amplification, followed by hybridization with genotype-specific probes capable of identifying most HPV genotypes in the genital region: 13 high-risk HPV genotypes (16, 18, 31, 33, 35, 39, 45, 51, 52, 53, 56, 58, 58, and 59), 11 low-risk genotypes (6, 11, 40, 43, 44, 54, 70, 66, 68, 73, and 82), and 3 indeterminate-risk genotypes (69, 71, and 74). The commercial method used was the NucliSenseasy MAG system (bioMérieux SA, Marct l’Etoile, France). The technique used has been described previously.

### 2.2. Surgical Procedures

For endocervical curettage, performed before LEEP, a Kevorkian curette was used. LEEP was performed with electrodes of varying shapes and sizes to fit the cervix to be treated. After the application of Lugol to assess the extent of cervical lesions and estimate the circumference and width of excision, we removed the entire atypical transformation zone with a margin of 3–5 mm outside. The length of the cone depends on the type of transformation zone. The type 1 transformation zone with a length of 1 cm was removed from the cervix. At least 1.5 cm and 2 cm were removed for TZ types 2 and 3, respectively. Electrocoagulation with ball loop of the cruentate bed followed until hemostasis was achieved. After cervical conization, the specimen was marked at 12 o’clock, and each specimen was measured to determine length and width before fixation. Two pathologists confirmed all histopathological findings of the entire LEEP specimen. The surgical procedure consisted of a standardized total laparoscopic hysterectomy.

### 2.3. Statistical Analysis

Statistical analysis was performed using SPSS software version 17.0 (SPSS Inc., Chicago, IL, USA). Continuous variables were described as mean ± standard deviation (SD) or median and interquartile range (IQR), based on the distribution assessed by the Kolmogorov−Smirnov test. Categorical variables were expressed as absolute frequencies and percentages. For comparison between groups with and without residual disease (defined as the presence of CIN2+, AIS, or carcinoma), the following were used: Student’s *t* test for continuous variables with normal distribution, Mann−Whitney U test for continuous variables not normally distributed, and chi-square (χ^2^) test for categorical variables. The strength of association between categorical variables and the presence of residual disease was quantified by Cramér’s coefficient (Cramér’s V). Cramér’s V values were interpreted as follows: <0.1 = no or weak association, 0.1–0.3 = weak association, 0.3–0.5 = moderate association, >0.5 = strong association. For identification of independent predictive factors of residual disease, binary logistic regression was performed. Variables with significance *p* < 0.05 at bivariate analysis were entered into the multivariate model. The results were expressed as odds ratios (ORs) with 95% confidence intervals (95% CIs). To evaluate the accuracy of the multivariate predictive model, a Receiver Operating Characteristic (ROC) curve was constructed. The area under the curve (AUC) was calculated to measure the discriminative ability of the model: AUC = 0.5: no discrimination, AUC = 0.7–0.8: good discrimination, AUC > 0.8: excellent discrimination. The optimal clinical score cut-off was determined as the point on the ROC curve closest to the upper left corner, and the associated sensitivity and specificity were reported. A *p* value < 0.05 was considered statistically significant in all analyses.

## 3. Results

Of the 189 women enrolled for the study, 35 were excluded: 24 cases for non-diagnostic ECC, and 11 cases for marked stenosis of the cervical canal, so the study sample consisted of 154 women. Among the 154 women analyzed, residual disease was diagnosed in 38 cases (24.7%), including 13 CIN3, 7 CIN2, 3 AIS, 4 squamous microcarcinomas (FIGO IA1, depth ≤ 3 mm), and 3 invasive squamous cell carcinomas (FIGO ≥ IA2). The clinical characteristics of the 154 eligible patients are listed in Table 1.

Positive margins were present in 61 patients, of whom 28 (45.9%) had residual disease. Among the 93 women with negative margins, 11 (11.8%) still had residual disease. Persistence of high-risk HPV after LEEP was found to be strongly associated with residual disease: 15 of 26 cases with HPV 16/18 post-LEEP (57.7%) had residual disease, as did 22 of 38 women with non-16/18 high-risk HPV genotypes (57.9%) (Table 1).

ECC outcome also showed a clear correlation with residual disease: among the 56 women with positive ECC, 27 (48.2%) had residual, while among the 67 with negative ECC, and only 9 (13.4%) had residual disease. This finding also showed a statistically significant association (*p* < 0.001) and significant predictive value in multivariate analysis (Table 2).

**Figure 1:**
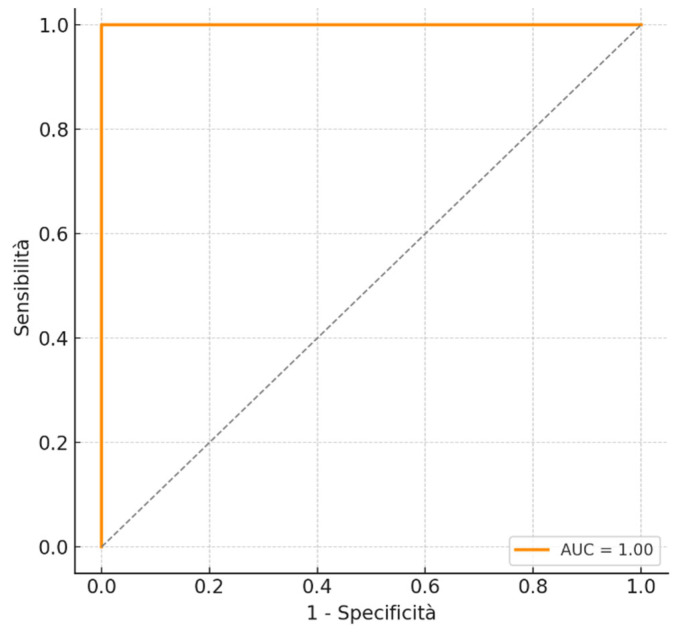
The ROC curve showed good accuracy, with an area under the curve (AUC) of 0.86.

To evaluate the discriminative ability of the multivariate model consisting of ECC positive, HPV 16/18 post-LEEP, and HPV non-16/18 high-risk post-LEEP, a Receiver Operating Characteristic (ROC) curve was constructed. The curve showed good accuracy, with an area under the curve (AUC) of 0.86. This value indicates that the model can effectively distinguish between patients with and without residual disease after LEEP. The high AUC reinforces the clinical value of the model in risk stratification and selection of patients who are candidates for hysterectomy (Figure 1).

## 4. Discussion

We observed residual disease in 25.3% (39/154) of postmenopausal women who underwent LEEP for CIN3 and then hysterectomy.

Data on recurrence or residual disease vary widely in the literature, as many studies do not clearly define recurrence and residual disease and often use the generic term “recurrent disease” without distinguishing between “recurrence” and “residual disease”, which leads to potentially vague interpretations.

In our study, a woman was considered to have residual disease after LEEP if the histological diagnosis still showed CIN2+, while recurrence was defined by the presence of at least one negative test between the initial LEEP and the diagnosis of recurrent CIN2+.

To reduce the bias related to the risk of progression or recurrence of CIN, we included only women with an interval of less than 12 months between LEEP and hysterectomy. At the same time, this study highlights how 115 women (74.7%), without residual disease, underwent unnecessary hysterectomy; excluding glandular pathology, for which the guidelines already direct towards hysterectomy. Overall, it highlights that the management of postmenopausal women deserves special attention, since hysterectomy, which many gynecologists resort to, can lead to overtreatment on one side or undertreatment if postoperative histology reveals occult carcinoma. In fact, in clinical practice, approximately 10–11% of patients with CIN receive a histological diagnosis of invasive cancer already in the LEEP specimen compared to colposcopic cervical biopsy [20,21,22]. This risk is higher in postmenopausal women, in whom the decrease in estrogen levels, the retraction of the squamocolumnar junction, and the atrophic changes of the postmenopausal cervix make both colposcopy and cytology ineffective, as well as the performance of a LEEP, more complicated. The risk of occult carcinoma has led many gynecologists to resort to hysterectomy, some even directly (without LEEP) in postmenopausal women, without considering that hysterectomy as a therapeutic option for CIN3 should be performed only after definitively excluding an invasive tumor, so that the latter can be treated adequately. Furthermore, long-term population studies have shown that the risk of cervical cancer after conization for the treatment of CIN3 persists for at least 25 years, underlining the need for careful follow-up and the importance of detecting the presence of residual disease [13]. Awareness of the need for such long-term follow-up has led many women to refuse surveillance and instead request a hysterectomy, perceived as a definitive solution to the problem. A critical aspect of the present study is the assessment of the risk of occult carcinoma: in the literature, the incidence of invasive carcinoma at the time of hysterectomy has decreased from 12.9% in previous studies to 6.4% in more recent studies, and in our sample, it is 4.5% [23,24,25,26,27]. The incidence of occult carcinoma (7/154) in the present study is clinically significant and supports the indication for surgery in selected cases. A total of 74.7% (115/154) of patients who underwent hysterectomy showed no significant histological residuals, raising the issue of overtreatment. This finding reinforces the need for a cautious and selective approach, particularly in patients with multiple risk factors at the same time. Our analysis shows that there are some factors, such as persistent high-risk HPV infection and positive endocervical curettage (ECC), that can predict residual disease in hysterectomy specimens after LEEP in postmenopausal women diagnosed with CIN3. These two elements identify postmenopausal women who underwent LEEP for CIN3 to undergo hysterectomy.

The present study confirms that the risk associated with the persistence of HPV 16/18 post-LEEP is extremely high (Table 2), indicating that this parameter should be systematically considered in the postoperative evaluation, data confirmed by the literature [28]. The same principle also applies to postmenopausal women, with the additional concern that, in this population, attention should also be paid to the presence of high-risk HPV genotypes other than 16/18 [29,30]. In fact, the results of the study highlight that even high-risk HPV genotypes other than 16/18 show a significant correlation with the presence of residual lesions, although with a wider confidence interval, confirming the potential biological heterogeneity of viral strains and the usefulness of extended genotyping in postmenopausal women [31,32,33].

The oncogenic potential of non-16/18 genotypes has been less studied in the literature, since non-16/18 hr HPV genotypes are often tested as a pool (partial genotyping). The use of extended HPV genotyping would highlight specific non-HPV 16/18 genotypes exploring the CIN2+ risk of each individual genotype.

In the literature, the predictive value and diagnostic accuracy of ECC remain vague due to limited studies [34,35,36,37]. The value of ECC lies in its ability to identify non-visible endocervical lesions, especially in the presence of a type 3 transformation zone, a condition frequently observed in postmenopausal women due to retraction of the squamocolumnar junction into the endocervical canal. Positive ECC has shown a significant association with the presence of residual disease.

The risk of incomplete excision of CIN2+ in postmenopausal women is significantly higher than in women of reproductive age. A type 3 transformation zone makes it difficult to ensure complete excision of the CIN2+ lesion during conization. It is difficult to adjust the depth and width of the transformation zone. It is not visible colposcopically and is difficult to reach with standard LEEP. In this context, ECC becomes the only tool capable of collecting cells from the deepest and most invisible area. Furthermore, the cervix in postmenopausal women is often very small, limiting the feasibility of biopsies, ECC, and deep conizations, thus increasing the risk that part of the lesion remains unexcised or unidentified. The situation is further complicated by the frequent presence of “skip” lesions, i.e., non-contiguous lesions located deep in the cervix (as observed in three case of our study). This could explain why nine cases of residual lesions were diagnosed despite negative ECC results.

Regarding cone margins, our study confirms a previously known but controversial finding: although positive margins are significantly associated with residual disease in the univariate analysis, they lose significance in the multivariate model, indicating that this factor alone is not sufficient to determine the indication for hysterectomy. This result is consistent with previous studies, which demonstrate that positive margins are a sensitive but non-specific indicator of residual disease [38,39].

The results of the ROC curve built on the multivariate model—including positive ECC, post-LEEP HPV 16/18, and high-risk non-16/18 HPV genotypes—further confirm the clinical value of these factors. With an area under the curve (AUC) of 0.86, the model demonstrates a good discriminatory ability in distinguishing between patients with and without residual disease. This value reinforces the practical utility of the tool, suggesting that it can be effectively used for risk stratification and to support personalized clinical decision-making. In particular, an AUC > 0.8 is considered indicative of high diagnostic accuracy and predictive validity.

An additional strength of our study is the use of the Cramér coefficient of association (Cramér’s V), which was used to assess the strength of the association between each categorical variable and the presence of residual disease. The highest Cramér’s V values were observed for post-LEEP HPV (0.630), ECC (0.381), and margin status (0.383), confirming that these are clinically and statistically significant predictors. In particular, the very high value for post-LEEP HPV suggests a strong association with the risk of residual disease.

Women treated for CIN have a significantly higher risk of subsequent diagnosis of cervical cancer and other HPV-related cancers than the general population. The elevated risk of cervical cancer persists for at least 20 years after treatment and is highest in women over the age of 50. Extended follow-up beyond the last screening cycle may be warranted for previously treated women [40].

Our analysis, therefore, suggests that hysterectomy after LEEP for CIN3 in postmenopausal women could be considered in the presence of a combination of high-risk factors: positive ECC, persistent HPV infection, and type 3 transformation zone. In the absence of these factors, close follow-up may be preferable, reducing the impact of major surgical intervention on often frail and comorbid women.

Among the main limitations of our study are its retrospective nature and the lack of centralized histological review, although data collection was standardized across both centers. Nevertheless, the sample size, the specificity of the target population (postmenopausal women with CIN3), and the use of Cramér’s V association coefficient provide robustness to the results.

## 5. Conclusions

This study demonstrates that the identification of specific risk factors (persistent high-risk HPV, positive ECC, and type 3 transformation zone) enables more accurate risk stratification for residual disease after LEEP for cervical CIN3 in postmenopausal women. Hysterectomy should be reserved for patients with combinations of these risk factors, in order to avoid overtreatment and ensure safer, more personalized management.

## Figures and Tables

**Table 1:** Key population characteristics and bivariate analysis (N = 154).

Variable	Total N (%)	Positive Residual N (%)	Negative Residual N (%)	*p*-Value	Cramer’s V
Negative Margins	93 (60.4)	11 (28.2)	82 (71.3)	<0.001	0.383
Positive Margins	61 (39.6)	28 (71.8)	33 (28.7)		
Negative HPV post-LEEP	90 (58.4)	2 (5.1)	88 (76.5)	<0.001	0.630
HPV no 16/18	38 (24.7)	22 (56.4)	16 (13.9)		
HPV 16/18	26 (16.9)	15 (38.5)	11 (9.6)		
Negative ECC	67 (54.5)	9 (25.0)	58 (66.7)	<0.001	0.381
Positive ECC	56 (45.5)	27 (75.0)	29 (33.3)		
TZ type 3	115 (74.7)	31 (79.5)	84 (73.0)	0.273	0.130

**Table 2:** Crude and adjusted odds ratios for independent risk factors.

Variable	Crude OR (IC 95%)	*p*-Value	Adjusted OR (IC 95%)	*p*-Value
Positive Margins	6.325 (2.825–14)	<0.001	1.757 (0.519–5.952)	0.365
Non-HPV 16/18 post-LEEP	60 (12–298)	<0.001	68 (7.8–610)	<0.001
HPV 16/18 post-LEEP	60 (13–283)	<0.001	74 (8.0–694)	<0.001
Positive ECC	6.000 (2.498–14)	<0.001	3.642 (1.154–11)	0.028

## Data Availability

The data are contained within the article.
